# ROCK1 Deficiency Enhances Protective Effects of Antioxidants against Apoptosis and Cell Detachment

**DOI:** 10.1371/journal.pone.0090758

**Published:** 2014-03-04

**Authors:** Michelle Surma, Caitlin Handy, Jiang Chang, Reuben Kapur, Lei Wei, Jianjian Shi

**Affiliations:** 1 Riley Heart Research Center, Indiana University, School of Medicine, Indianapolis, Indiana, United States of America; 2 Herman B Wells Center for Pediatric Research, Department of Pediatrics, Indiana University, School of Medicine, Indianapolis, Indiana, United States of America; 3 Texas A&M University Health Science Center, Institute of Biosciences and Technology, Houston, Texas, United States of America; University of Birmingham, United Kingdom

## Abstract

We have recently reported that the homologous Rho kinases, ROCK1 and ROCK2, play different roles in regulating stress-induced stress fiber disassembly and cell detachment, and the ROCK1 deficiency in mouse embryonic fibroblasts (MEF) has remarkable anti-apoptotic, anti-detachment and pro-survival effects against doxorubicin, a chemotherapeutic drug. This study investigated the roles of ROCK isoforms in doxorubicin-induced reactive oxygen species (ROS) generation which is believed to be the major mechanism underlying its cytotoxicity to normal cells, and especially to cardiomyocytes. Different antioxidants have been shown to provide a protective role reported in numerous experimental studies, but clinical trials of antioxidant therapy showed insufficient benefit against the cardiac side effect. We found that both *ROCK1^−/−^* and *ROCK2^−/−^* MEFs exhibited reduced ROS production in response to doxorubicin treatment. Interestingly, only ROCK1 deficiency, but not ROCK2 deficiency, significantly enhanced the protective effects of antioxidants against doxorubicin-induced cytotoxicity. First, ROCK1 deficiency and N-acetylcysteine (an anti-oxidant) treatment synergistically reduced ROS levels, caspase activation and cell detachment. In addition, the reduction of ROS generation in *ROCK1^−/−^* MEFs in response to doxorubicin treatment was in part through inhibiting NADPH oxidase activity. Furthermore, ROCK1 deficiency enhanced the inhibitory effects of diphenyleneiodonium (an inhibitor of NADPH oxidase) on ROS generation and caspase 3 activation induced by doxorubicin. Finally, ROCK1 deficiency had greater protective effects than antioxidant treatment, especially on reducing actin cytoskeleton remodeling. ROCK1 deficiency not only reduced actomyosin contraction but also preserved central stress fiber stability, whereas antioxidant treatment only reduced actomyosin contraction without preserving central stress fibers. These results reveal a novel strategy to enhance the protective effect of antioxidant therapy by targeting the ROCK1 pathway to stabilize the actin cytoskeleton and boost the inhibitory effects on ROS production, apoptosis and cell detachment.

## Introduction

The ROCK family contains two members: ROCK1 and ROCK2, which share 92% identity in the kinase domain [1]–[3]. ROCK plays a critical role in mediating the effects of small GTPase RhoA on stress fiber formation, smooth muscle contraction, cell adhesion, and cell motility [4], [5]. The best characterized targets of ROCK in the vascular system are myosin light chain (MLC) phosphatase (MYPT), MLC [6]–[8], and LIM-kinases (LIMK) [9], [10]. These ROCK-mediated pathways are known to be involved in stress fiber assembly and cell adhesion. Using mouse embryonic fibroblasts (MEFs) derived from *ROCK1^−/−^* and *ROCK2^−/−^* mice, we recently demonstrated that ROCK1 and ROCK2 can differently regulate stress fiber disassembly and cell adhesion under stress conditions such as cytotoxicity induced by doxorubicin, a chemotherapeutic agent used to treat a wide spectrum of hematologic malignancies and solid tumors for decades, or serum starvation, a frequently used environmental stress in cell biology [11], [12]. We demonstrated that ROCK2 is required for stabilizing the actin cytoskeleton through regulating cofilin phosphorylation under stress conditions, and in contrast, ROCK1 is involved in destabilizing the actin cytoskeleton through regulating MLC phosphorylation and peripheral actomyosin contraction [11], [12]. These findings support a novel concept that ROCK1 and ROCK2 can differently regulate stress fiber disassembly, cell adhesion and cell death under stress conditions.

Although doxorubicin has been shown to increase actin cytoskeleton instability through the inhibition of actin polymerization [13], [14], the excessive generation of free reactive oxygen species (ROS) in the drug treatment can also be involved in actin cytoskeleton remodeling and promoting the activation of multiple signaling mechanisms, resulting in cell damage via apoptosis, necrosis, autophagy and senescence [15]–[22]. ROS production is believed to be the major mechanism underlying its cytotoxicity to normal cells in most major organs, including heart [20]–[26]. Many studies have suggested that antioxidants have a protective effect on chemotherapy-induced cytotoxicity. However, clinical trials of antioxidant therapy showed only limited beneficial effects [27], [28].

In this study, we showed that the deletion of either isoforms of ROCK inhibited ROS production induced by doxorubicin. Interestingly, only ROCK1 deficiency, but not ROCK2 deficiency, significantly enhanced the protective effects of antioxidants against doxorubicin-induced cytotoxicity. We also showed that the reduction of ROS generation in *ROCK1^−/−^* MEFs under doxorubicin treatment was in part through inhibiting NADPH oxidase activity. ROCK1 deficiency and N-acetylcysteine (NAC, an anti-oxidant) treatment synergistically reduced ROS levels, caspase activation and cell detachment. We further showed that ROCK1 deficiency enhanced the inhibitory effects of diphenyleneiodonium (DPI, an inhibitor of NADPH oxidase) on ROS generation and caspase 3 activation. Importantly, ROCK1 deficiency had greater effects than antioxidant treatment in reducing actin cytoskeleton remodeling. Our results suggest that the greater anti-remodeling effects of ROCK1 deficiency are mediated through reducing actomyosin contraction and preserving central stress fiber stability; NAC treatment, though it also decreases actomyosin contraction, does not stabilize central stress fibers, therefore providing less protection to actin cytoskeleton structure. These results reveal a novel strategy to improve antioxidant therapeutic efficacy by adding a ROCK isoform specific inhibitor to target the ROCK1 pathway. Their synergistic effects may further extend the protection against oxidative stress through stabilizing the actin cytoskeleton, decreasing ROS overproduction and apoptosis, improving cell adhesion as well as alleviating tissue remodeling.

## Results

### ROCK1 Deletion Reduces Doxorubicin-induced ROS Production

**Figure 1:**
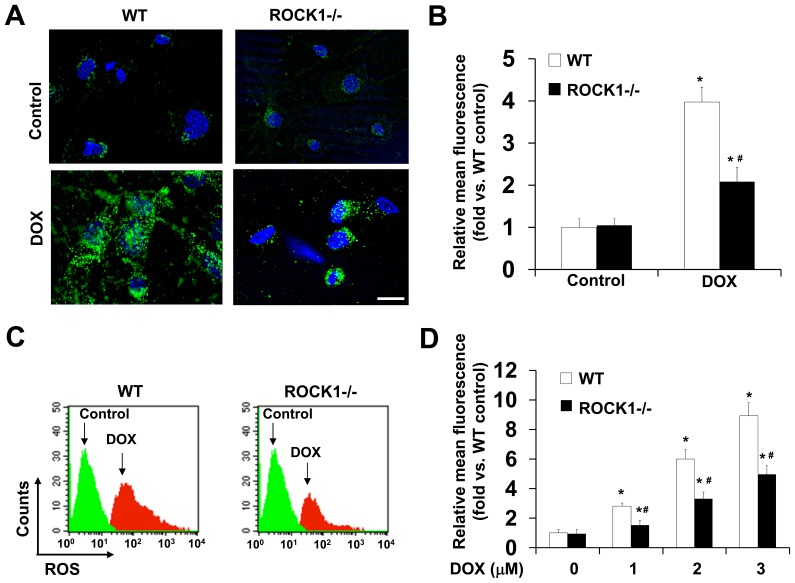
ROCK1 deletion reduces doxorubicin-induced ROS production. A. Representative images of CM-H2DCFDA staining. WT and *ROCK1^−/−^* MEFs were grown on glass coverslips, treated for 4 h with 3 µM doxorubicin and then exposed to 13 µM CM-H2DCFDA. Coverslip was mounted with Vector mounting medium containing DAPI and imaged immediately. Bar, 50 µm. B. Quantitative analysis of the images obtained from fluorescence microscopy with the Leica AF6000 software. Fluorescence levels in WT cells at baseline were arbitrarily set at 1. C and D. Representative results (C) and quantitative analysis (D) of ROS measured by flow cytometry analysis after staining with CM-H2DCFDA. WT and *ROCK1^−/−^* cells were treated for 16 h with 3 µM doxorubicin (C) or with the dosages of doxorubicin as indicated (D). The attached cells were stained with 5 µM CM-H2DCFDA, collected and analyzed by flow cytometry in at least three separate experiments. Fluorescence levels in WT cells at baseline were arbitrarily set at 1. *P<0.05 vs. control of the same genotype. ^#^ *P*<0.05 vs. WT under the same treatment condition.

Free radical generation and oxidative stress occur soon after starting doxorubicin treatment and significantly contribute to the drug’s cytotoxic effects. Our previous studies revealed that the deletion of ROCK1 in MEFs inhibits apoptosis and cell detachment [11], [12]. To determine if the protective effects of ROCK1 deficiency contributes to the reduced ROS, we measured intracellular ROS generation with the chloromethyl derivative of dichlorodihydrofluorescein diacetate (CM-H2DCFDA), an oxidant-sensitive dye (Fig. 1). As expected, fluorescence microscopy analysis revealed that ROS levels increased by 3.97 folds in WT MEFs in response to 3 µM of doxorubicin treatment for 4 h (Fig. 1A–B). Interestingly, ROS generation was markedly reduced in ROCK1 deficient MEFs (2.08-fold increase, Fig. 1A–B). The doxorubicin-induced ROS overproduction was also observed by flow cytometry in WT MEFs in a dose-dependent manner (Fig. 1C–D). This dose-dependent increase of ROS was significantly reduced in ROCK1 deficient MEFs (Fig. 1C–D). These results suggest that the inhibition of intracellular ROS by ROCK1 deletion contributes, at least in part, to its cytoprotective effects.

### ROCK1 Deletion Enhances Anti-ROS, Pro-survival, Anti-apoptotic Effects of the Anti-oxidant NAC

**Figure 2:**
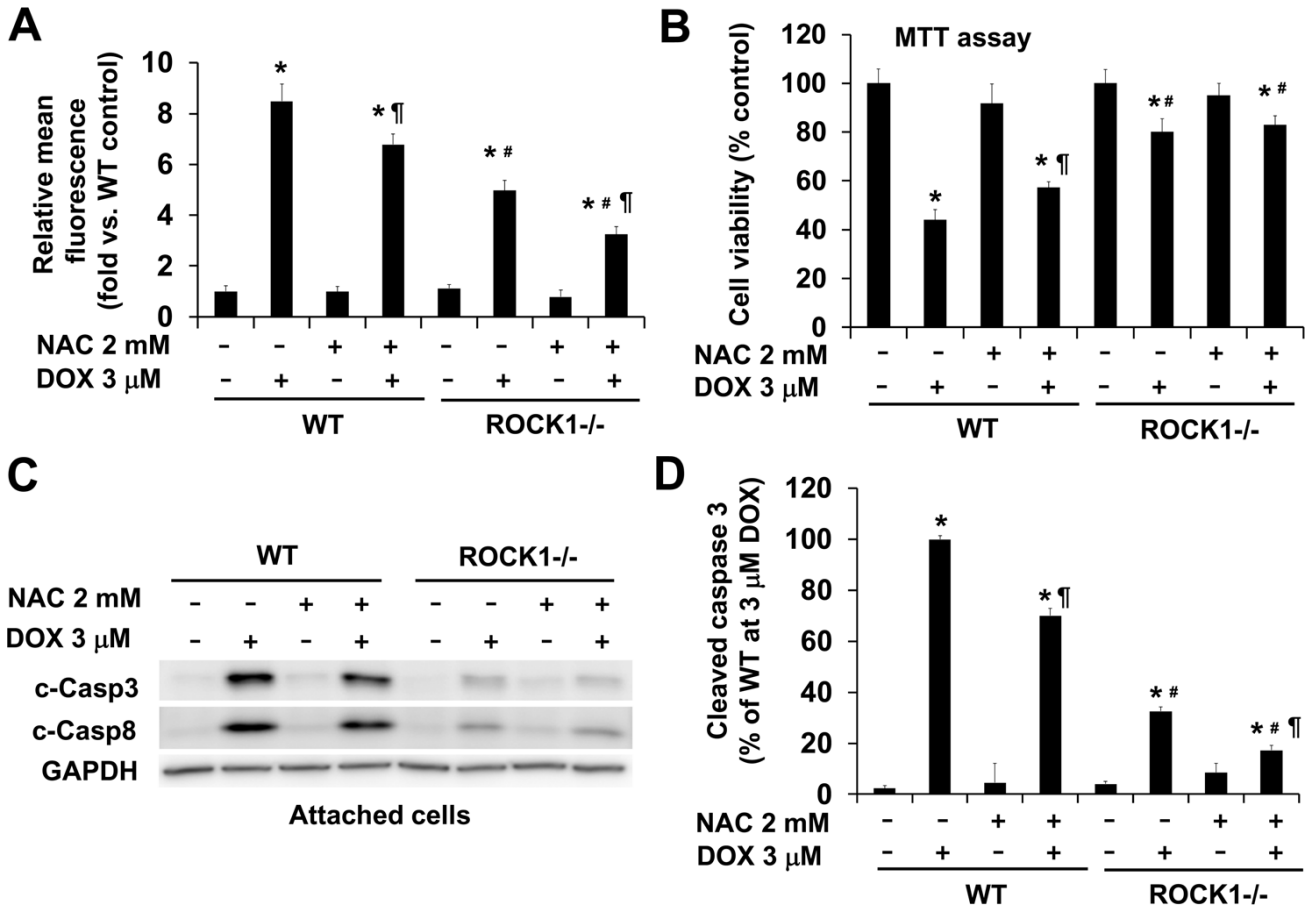
ROCK1 deletion enhances anti-ROS, pro-survival, anti-apoptotic effects of NAC. A. ROS levels measured by flow cytometry analysis after CM-H2DCFDA staining of attached WT and *ROCK1^−/−^* MEFs after 3 µM doxorubicin and/or 2 mM NAC treatment for 16 h; showing a minimal doxorubicin-induced ROS production in NAC treated ROCK1 deficient MEFs. Fluorescence levels in WT cells at baseline were arbitrarily set at 1. B. MTT assay performed with MEFs treated for 16 h with 3 µM doxorubicin and/or 2 mM NAC showing maximal level of cell viability after doxorubicin treatment in NAC treated ROCK1 deficient MEFs. C. Representative image of Western blot of cleaved caspase-3 and -8 in cell lysates from attached WT and ROCK1 deficient MEFs treated with 3 µM doxorubicin and/or 2 mM NAC for 16 h. Equal amount of proteins were loaded. D. Densitometry analysis of immunoreactive bands of cleaved caspase-3 expressed as percent change relative to WT cells treated with 3 µM doxorubicin, showing a minimal level of caspase activation in NAC treated ROCK1 deficient MEFs. *P<0.05 vs. control of the same genotype. ^#^ *P*<0.05 vs. WT under the same treatment condition. ^¶^ *P*<0.05 vs. the same genotype under doxorubicin only condition.

NAC has multiple clinical applications attributed to its ability to support the body’s antioxidant and nitric oxide systems during stress, infections, toxic assault, and inflammatory conditions due to its fast reactions with free radicals [29], [30]. In addition, it protects animals against doxorubicin-induced cardiotoxicity [31]. As expected, the treatment of WT MEFs with 2 mM of NAC inhibited doxorubicin-induced ROS production measured by flow cytometry (Fig. 2A). In addition, the improved viability revealed by MTT assay (Fig. 2B) and reduced doxorubicin-induced caspase activation (Fig. 2C–D) were also observed in NAC treated WT MEFs. It is worth noting that the protective effect of 2 mM of NAC was less effective than the effect of ROCK1 deletion (Fig. 2A–D). Besides the treatment with 2 mM of NAC and 3 µM of doxorubicin for 16 h, which was used for most of the experiments presented in this study, we have also analyzed the cells with different drug concentrations, for example, different NAC concentrations ranging from 1 to 10 mM, doxorubicin concentrations varying from 1 to 5 µM, and time points changing from 4 to 24 h. We found that under all tested conditions, the anti-ROS and anti-apoptotic effects of NAC treatment were less effective than those from ROCK1 deletion (data not shown). Moreover, synergistic effects of NAC treatment and ROCK1 deletion were evidenced by the further reduction of doxorubicin-induced ROS production (Fig. 2A) and caspase activation (Fig. 2C–D) in NAC treated ROCK1 deficient MEFs. A trend of an increase in cell viability was also observed in NAC treated ROCK1 deficient MEFs, but the difference was not statistically significant (Fig. 2B). Therefore, NAC treatment together with ROCK1 deletion was more effective than NAC treatment or ROCK1 deletion alone on reducing ROS production and suppressing caspase activation.

### ROCK1 Deletion Reduces Doxorubicin Induced Actin Cytoskeleton Remodeling to a Greater Degree than Antioxidant NAC

**Figure 3:**
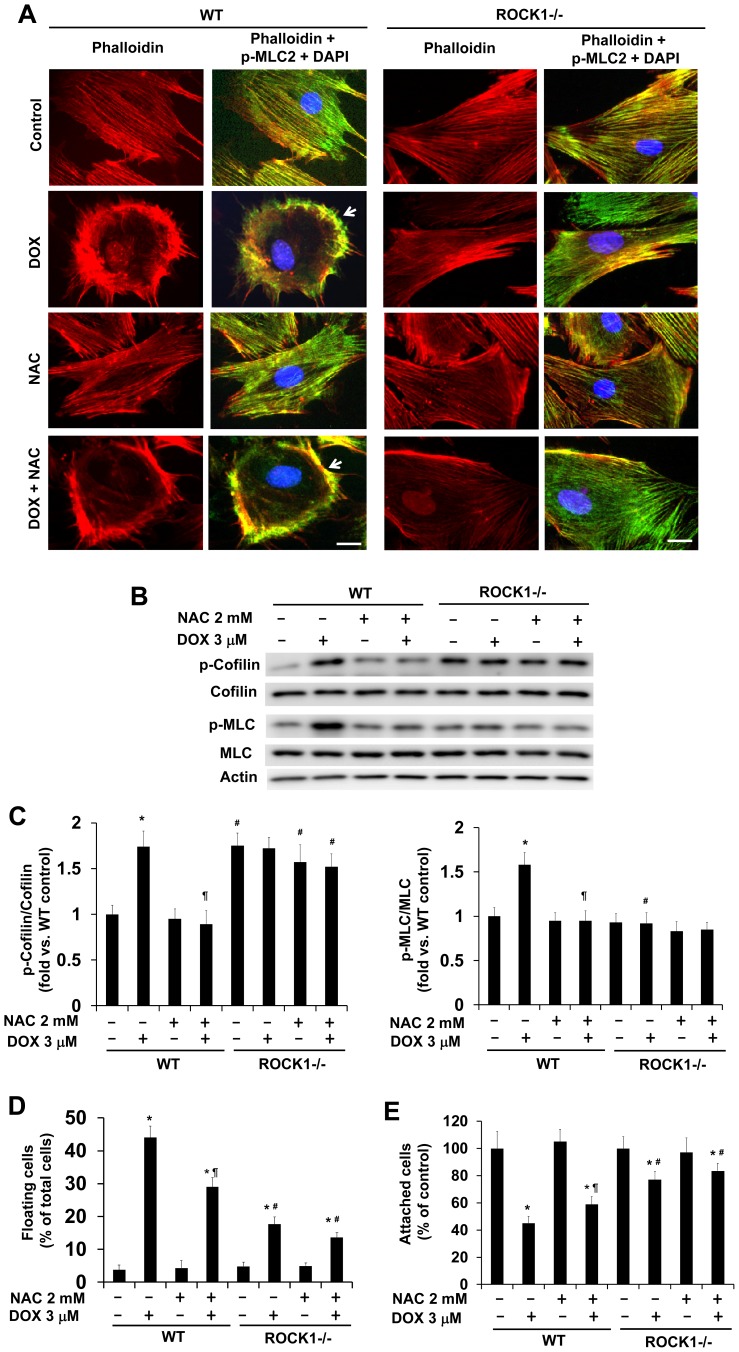
ROCK1 deletion enhances anti-detachment effects of NAC. A. Representative images of rhodamine-phalloidin staining for F- actin (red), p-MLC staining (green) and DAPI (blue) of WT and ROCK1 deficient cells treated with 3 µM doxorubicin and/or 2 mM NAC for 8 h. Cells showing disruption of central stress fibers are indicated with white arrowheads. ROCK1 deletion, but not NAC treatment, prevents DOX-induced disruption of central stress fibers. Bar, 25 µm. B and C. Representative image (B) of Western blot of p-MLC2 and MLC2, or p-cofilin and cofilin in cell lysates from attached WT and *ROCK1^−/−^* MEFs treated with 3 µM doxorubicin and/or 2 mM NAC for 30 min. Quantitative analysis (C) showing reduction of both p-MLC and p-cofilin in WT MEFs after NAC/DOX treatment compared to DOX treatment alone. The ratio of p-MLC to MLC or p-cofilin to cofilin was expressed as fold change relative to WT control. D and E. Both floating and attached cells were collected after treatment for 16 h with 3 µM doxorubicin and/or 2 mM NAC. Floating cell ratio was expressed as percentage of total cells (floating plus attached cells) under each treatment condition (D). Attached cell number was expressed as percentage of attached cells under control condition without treatment (E). *P<0.05 vs. control of the same genotype. ^#^ *P*<0.05 vs. WT under the same treatment condition. ^¶^ *P*<0.05 vs. the same genotype under doxorubicin only condition.

Our previous study [11], [12] revealed that doxorubicin induces actin cytoskeleton remodeling which is characterized by the formation of a cortical contractile ring at the cell periphery accompanied by the disruption of central stress fibers as shown in Fig. 3A. It induces an increase in MLC phosphorylation, which mediated by both ROCK1 and ROCK2, and contributes to the increased actomyosin contraction; it also induces an increase in cofilin phosphorylation which mainly mediated by ROCK2, promotes actin polymerization and stress fiber stability [11], [12].

To evaluate the effects of NAC treatment on actin cytoskeleton remodeling, we examined actin cytoskeleton changes after doxorubicin treatment in the presence and absence of NAC. Phalloidin staining revealed that the NAC treatment did not prevent doxorubicin-induced disruption of central stress fibers (Fig. 3A) and had an inhibitory effect on doxorubicin-induced cofilin phosphorylation (Fig. 3B–C). However, NAC treatment also had inhibitory effects on doxorubicin-induced MLC phosphorylation (Fig. 3B–C). Our previous study using blebbistatin, a direct inhibitor of myosin II ATPase, revealed that doxorubicin-induced actomyosin contraction contributes to cell detachment [11]. Consistent with reduced MLC phosphorylation, NAC treatment decreased doxorubicin-induced cell detachment (Fig. 3D–E). Compared to NAC treatment, ROCK1 deletion was more effective on reducing actin cytoskeleton remodeling and cell detachment induced by doxorubicin (Fig. 3A–E). ROCK1 deletion not only reduced doxorubicin-induced MLC phosphorylation but also preserved cofilin phosphorylation (Fig. 3B–C) as previously observed [11], [12]. Similar to the anti-ROS and anti-apoptotic effects described above, NAC treatments at the concentrations ranging from 1 to 10 mM were all less effective than ROCK1 deletion on reducing doxorubicin-induced cell detachment (data not shown). The NAC treatment in ROCK1 deficient MEFs showed a trend of further inhibiting cell detachment, but the difference was not statistically significant (Fig. 3D–E). Consistent with preserved actin cytoskeleton stability in ROCK1 deficient MEFs, co-treatment of doxorubicin with NAC had no detectable effects on MLC and cofilin phosphorylation in these cells (Fig. 3B). These results support the superior ability of ROCK1 deletion in preserving actin cytoskeleton stability, which underlies the synergistic effects of ROCK1 deletion and antioxidant treatment against doxorubicin-induced cytotoxicity.

### ROCK1 Deletion Reduces NADPH Oxidase Activation Induced by Doxorubicin

**Figure 4:**
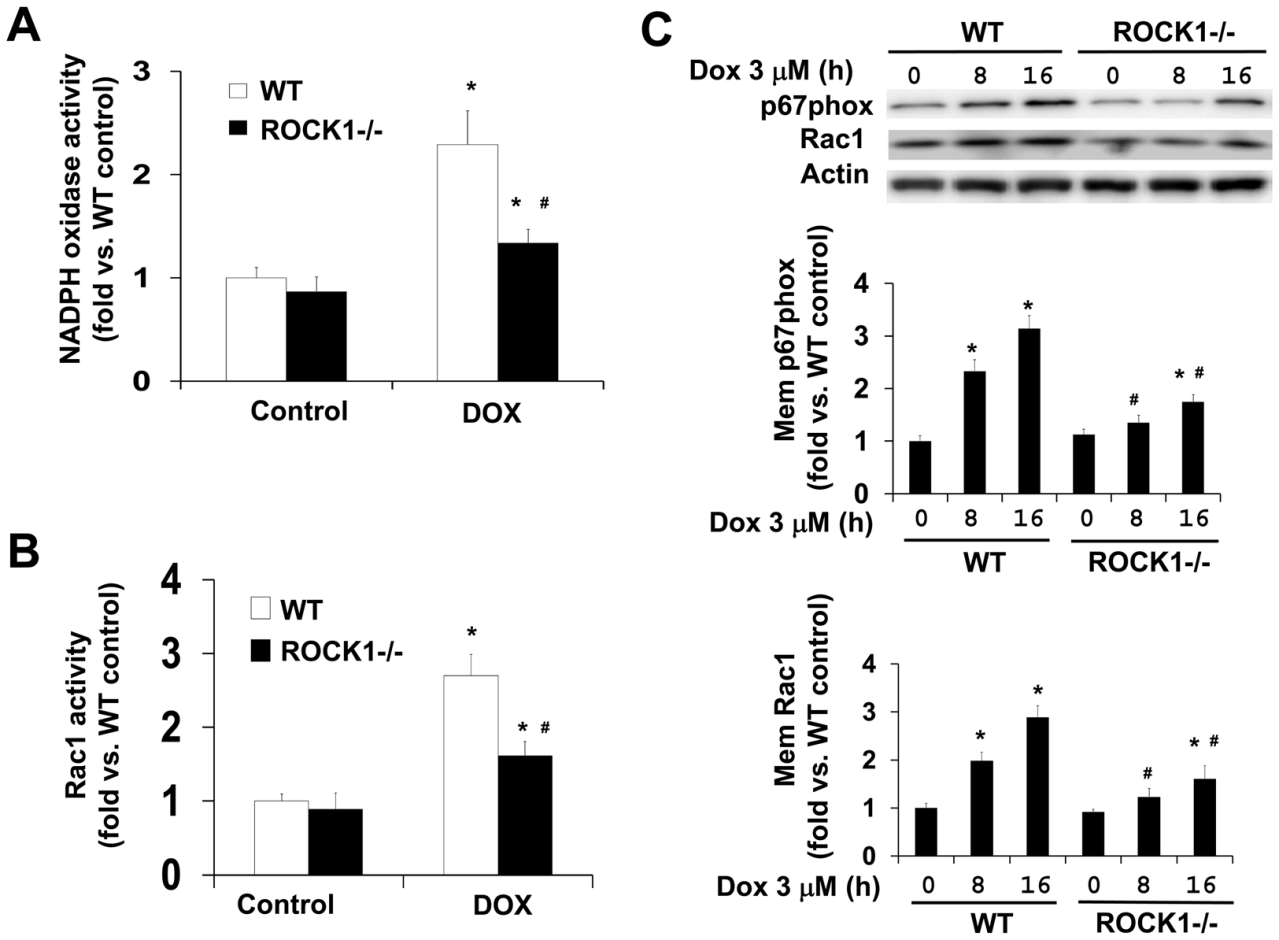
ROCK1 deletion reduces NADPH oxidase and Rac1 activity and membrane translocation of p67phox and Rac1 in response to doxorubicin. NADPH oxidase activity assay (A) and Rac1 activity pull-down assay (B) were performed with WT and *ROCK1^−/−^* MEFs after treatment for 16 h with 3 µM doxorubicin showing reduced NADPH oxidase activation in ROCK1 deficient cells. The activity was expressed as fold change relative to WT control. C. Representative image (top) and quantitative analysis (bottom) of Western blot of p67phox and Rac1 performed with membrane fractions from attached WT and *ROCK1^−/−^* MEFs treated with 3 µM of doxorubicin at indicated time points. *P<0.05 vs. control of the same genotype. ^#^ *P*<0.05 vs. WT under the same treatment condition.

NADPH oxidases have been identified as important sources of doxorubicin-induced ROS production [32], [33]. The prototypic NADPH oxidase comprises a catalytic core (Nox2/p22phox) and several cytosolic subunits (p47phox, p67phox, p40phox, Rac), which associate together upon activation [34]. Since ROCK1 deletion alone or allied with NAC inhibited doxorubicin-induced ROS production (Fig. 1), we asked if ROCK1 deficiency affects NADPH oxidase activity. We measured NADPH oxidase activity in WT and *ROCK1^−/−^* MEFs before and after doxorubicin treatment (Fig. 4A). The results indicated that doxorubicin treatment increased NADPH oxidase activity in WT MEFs, but this drug-induced NADPH oxidase activity was markedly reduced in ROCK1 deficient MEFs (Fig. 4A). Rac1 is known as an important subunit of NADPH oxidase and its activation is essential for the oxidase activity [35], [36]. To examine if Rac1 activation is involved in doxorubicin-induced cytotoxicity, we measured Rac1 activity through affinity precipitation of active Rac1 (Rac1-GTP) using GST fusion-protein containing the p21-binding domain (PBD) of Rac1 effector, p21-activated protein kinase (PAK)-1, followed by Western blot analysis. The results indicate that doxorubicin treatment increased Rac1 activity which is consistent to the increased NADPH oxidase activity in WT MEFs, but the activity was markedly reduced in ROCK1 deficient MEFs (Fig. 4B). Coinciding with doxorubicin-elevated NADPH oxidase and Rac1 activations, membrane translocations of both p67phox and Rac1 were found increased in WT MEFs, but were significantly reduced in ROCK1 deficient MEFs (Fig. 4C).

### ROCK1 Deletion Enhances the Anti-ROS and Anti-apoptotic Effects of NADPH Oxidase Inhibitor DPI

**Figure 5:**
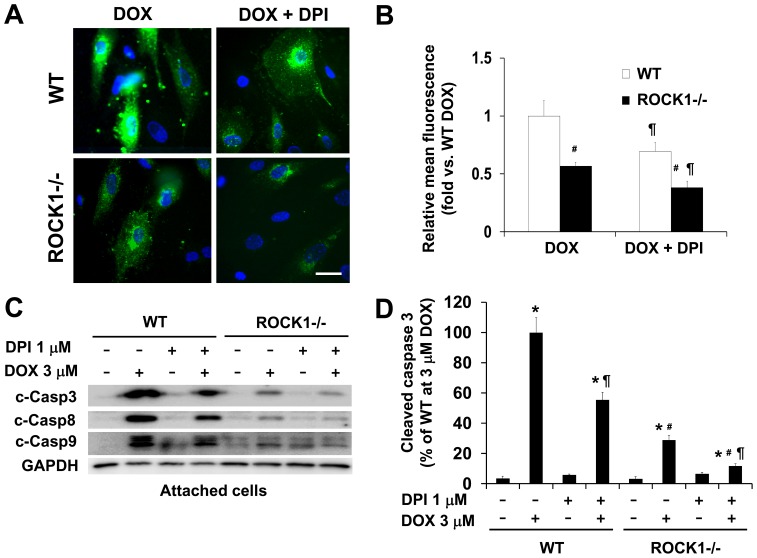
ROCK1 deletion enhances the anti-ROS and anti-apoptotic effects of DPI. A. Representative images of CM-H2DCFDA staining of WT and *ROCK1^−/−^* MEFs treated for 4 h with 3 µM doxorubicin and/or 1 µM DPI. Coverslip was mounted with Vector mounting medium containing DAPI and imaged immediately by fluorescence microscopy. Bar, 50 µm. B. Quantitative analysis of the images obtained from fluorescence microscopy with the Leica AF6000 software. Fluorescence level in WT cells under doxorubicin only condition was arbitrarily set at 1. C. Representative image of Western blot of cleaved caspase-3, -8, and -9 in cell lysates from attached WT and ROCK1 deficient MEFs treated for 16 h with 3 µM doxorubicin and/or 1 µM DPI. Equal amount of proteins were loaded. D. Densitometry analysis of immunoreactive bands of cleaved caspase-3 expressed as percent change relative to WT cells treated with 3 µM doxorubicin, showing minimal level of caspase activation in DPI treated ROCK1 deficient MEFs. *P<0.05 vs. control of the same genotype. ^#^ *P*<0.05 vs. WT under the same treatment condition. ^¶^ *P*<0.05 vs. the same genotype under doxorubicin only condition.

We subsequently examined the contribution of NADPH oxidase activity to doxorubicin-induced ROS generation and caspase activation using DPI, a chemical inhibitor. DPI treatment at 1 µM reduced doxorubicin-induced ROS generation in WT cells as well as in ROCK1 deficient cells (Fig. 5A–B). In addition, DPI treatment attenuated caspase activation in WT MEFs (Fig. 5C–D) and further reduced caspase activation in ROCK1 deficient MEFs, supporting synergistic protective effects of DPI and ROCK1 deletion. Moreover, increasing concentrations of DPI to 5–10 µM did not improve its anti-ROS and anti-apoptotic effects, but instead, showed increased cytotoxic effects with reduced cell viability (data not shown). Similar to the NAC treatment described above, under all tested conditions with different doses and time points, the protective effect of ROCK1 deletion was more effective than that of DPI (data not shown).

### ROCK1 Deletion Preserves Central Stress Fibers in DPI and Doxorubicin Treated Cells

**Figure 6:**
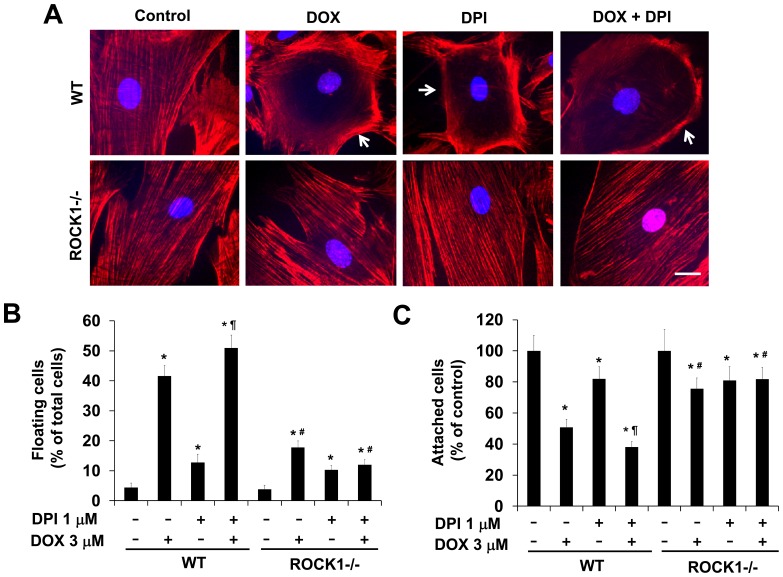
ROCK1 deletion preserves central stress fibers in DPI and doxorubicin treated cells. A. Representative images of rhodamine-phalloidin staining for F-actin (red) and DAPI staining (blue) of WT and *ROCK1^−/−^* cells treated with 3 µM doxorubicin and/or 1 µM DPI for 16 h. Cells showing disruption of central stress fibers are indicated with white arrowheads. DPI treatment alone induces disruption of central stress fibers in WT cells. ROCK1 deletion prevents doxorubicin and/or DPI-induced disruption of central stress fibers. Bar, 25 µm. B and C. DPI increases doxorubicin-induced cell detachment in WT MEFs but not in ROCK1 deficient MEFs. Floating cells (B) and attached cells (C) were collected at 16 h post-treatment with 3 µM doxorubicin and/or 1 µM DPI. Floating cell ratio was expressed as percentage of total cells (floating plus attached cells) under each treatment condition. Attached cell number was expressed as percentage of attached cells under control condition without treatment. *P<0.05 vs. control of the same genotype. ^#^ *P*<0.05 vs. WT under the same treatment condition. ^¶^ *P*<0.05 vs. the same genotype under doxorubicin only condition.

To examine the effects of DPI on doxorubicin-induced actin cytoskeleton remodeling, we performed phalloidin staining in WT and *ROCK1^−/−^* MEFs. We observed that DPI treatment alone induced the disruption of central stress fibers in WT MEFs (Fig. 6A). Although the mechanisms underlying this disruptive effect of DPI on the actin cytoskeleton are not clear, *ROCK1^−/−^* MEFs were resistant to this cytotoxic effect with preserved central stress fibers (Fig. 6A). Our previous study using cytochalasin D, an inhibitor of actin polymerization, indicated that the disruption of central stress fibers exaggerates doxorubicin-induced cell detachment [11]. Consistent with the apparent disruptive effects of DPI on the stress fibers in WT MEFs but not in *ROCK1^−/−^* MEFs (Fig. 6A), the co-treatment with DPI further worsened doxorubicin-induced cell detachment in WT MEFs but not in *ROCK1^−/−^* MEFs (Fig. 6B–C). Although *ROCK1^−/−^* MEFs were resistant to the disruptive effects of DPI on stress fibers, DPI treatment alone was able to cause a slight but significant increase in cell detachment (Fig. 6B–C), suggesting that DPI has multiple cytotoxic effects. Together, these results further support the concept that ROCK1 deletion is highly effective in inhibiting actin cytoskeleton remodeling induced by various cytotoxic stresses.

### ROCK2 Deletion does not Enhance Protective Effects of Antioxidants

**Figure 7:**
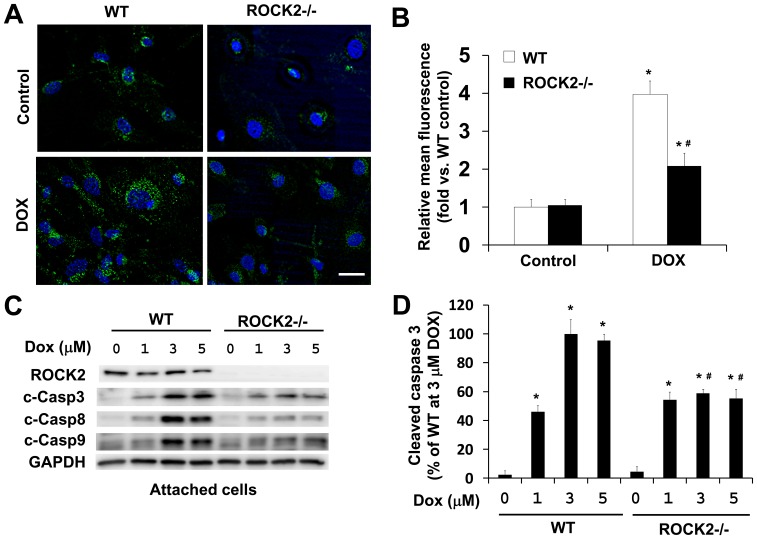
ROCK2 deletion reduces DOX-induced ROS production and caspase activation. A. Representative images of CM-H2DCFDA staining of WT and *ROCK2^−/−^* MEFs treated for 4 h with 3 µM of doxorubicin. Coverslip was mounted with Vector mounting medium containing DAPI and imaged immediately by fluorescence microscopy. Bar, 50 µm. B. Quantitative analysis of the images obtained from fluorescence microscopy with the Leica AF6000 software. Fluorescence levels in WT cells at baseline were arbitrarily set at 1, showing reduced doxorubicin-induced ROS production in ROCK2 deficient cells. C. Representative image of Western blot of ROCK2, cleaved caspase-3, -8, and -9 in cell lysates from attached WT and *ROCK2^−/−^* MEFs treated for 16 h with increasing dosages of doxorubicin as indicated. D. Quantitative analysis of immunoreactive bands of cleaved capsase-3 (*n* = 4–6 for each condition), expressed as percent change relative to WT cells treated with doxorubicin, showing reduced caspase activation in ROCK2 deficient cells treated with 3 or 5 µM doxorubicin. *P<0.05 vs. control of the same genotype.^ #^ *P*<0.05 vs. WT under the same treatment condition.

**Figure 8:**
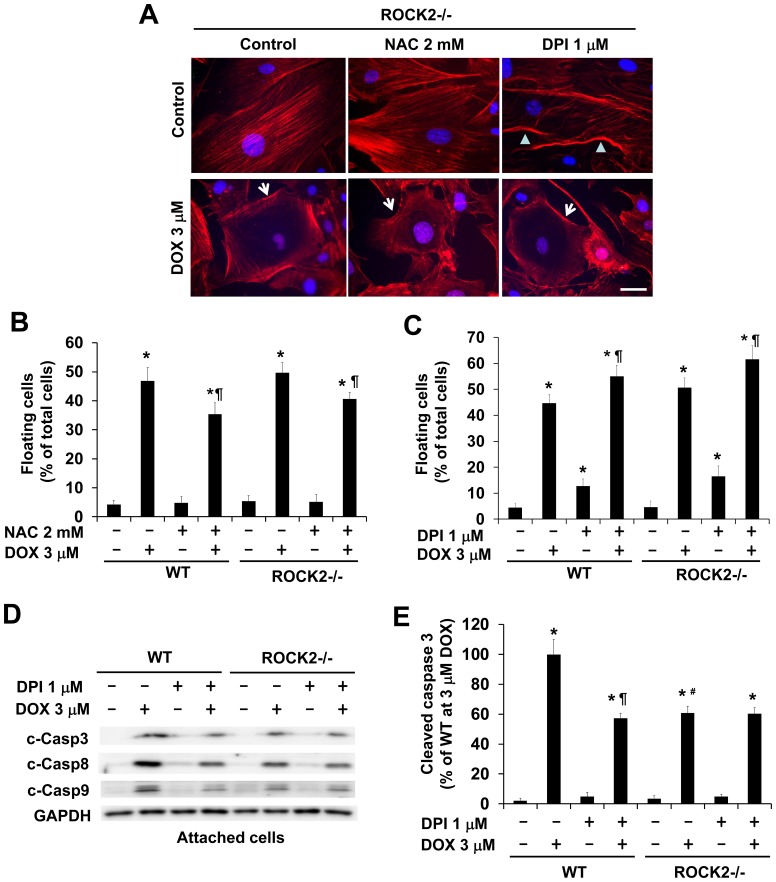
ROCK2 deletion does not preserve central stress fibers in doxorubicin-treated cells with or without NAC or DPI, and does not enhance the anti-apoptotic effect of DPI. A. Representative images of rhodamine-phalloidin staining for F-actin (red) and DAPI staining (blue) of *ROCK2^−/−^* cells treated with 3 µM doxorubicin and/or 2 mM NAC or 1 µM DPI for 16 h. Cells showing disruption of central stress fibers are indicated with white arrowheads. Cells showing folded periphery membranes are indicated with white arrowheads. DPI treatment alone induces periphery membrane folding in *ROCK2^−/−^* cells. Bar, 50 µm. B and C. Floating and attached cells were collected at 16 h post-treatment with 3 µM doxorubicin and/or 2 mM NAC (B) and/or 1 µM DPI (C). Floating cell ratio was expressed as percentage of total cells (floating plus attached cells) under each treatment condition. No significant difference was observed between WT and ROCK2 deficient cells under the same treatment condition. ROCK2 deletion does not enhance the anti-detachment effects of NAC. DPI treatment increases doxorubicin-induced cell detachment in WT and ROCK2 deficient MEFs. D. Representative image of Western blot of cleaved caspase-3, -8, and -9 in cell lysates from attached WT and ROCK2 deficient MEFs treated for 16 h with 3 µM doxorubicin and/or 1 µM DPI. E. Densitometry analysis of immunoreactive bands of cleaved caspase-3 expressed as percent change relative to WT cells treated with 3 µM doxorubicin, showing no further reduction in caspase activation by DPI treatment in doxorubicin-treated ROCK2 deficient MEFs. *P<0.05 vs. control of the same genotype. ^#^ *P*<0.05 vs. WT under the same treatment condition. ^¶^ *P*<0.05 vs. the same genotype under doxorubicin only condition.

To examine the impact of ROCK2 deletion on the protective effects of antioxidants, we assessed the effects of NAC or DPI on doxorubicin-induced cytotoxicity in *ROCK2^−/−^* MEFs. Similar to *ROCK1^−/−^* MEFs, *ROCK2^−/−^* MEFs also exhibited reduced ROS production in doxorubicin treatment (Fig. 7A–B), which was accompanied by reduced caspase activation (Fig. 7C–D). However, NAC treatment of *ROCK2^−/−^* MEFs had no additional anti-ROS and anti-apoptotic effects (data not shown). Resembling the finding in WT MEFs, NAC or DPI treatment in *ROCK2^−/−^* MEFs was not able to attenuate doxorubicin-induced disruption of central stress fibers (Fig. 8A). Moreover, DPI treatment alone increased peripheral membrane folding in *ROCK2^−/−^* MEFs (Fig. 8A), consistent with our previous observations that ROCK2 deletion in MEFs leads to increased actin cytoskeleton instability and impaired cell adhesion [11], [12]. In accordance with cell adhesion impairment, *ROCK2^−/−^* MEFs showed a trend toward increasing cell detachment compared to the WT MEFs after doxorubicin, with or without the co-treatment of NAC (Fig. 8B) or DPI (Fig. 8C). Besides, we did not find any additional effect of DPI treatment on decreasing caspase activation in *ROCK2^−/−^* MEFs (Fig. 8D–E). Together these results indicate that ROCK2 deletion, in contrast to ROCK1 deletion, does not enhance the cytoprotective effects of antioxidants, likely due to its deleterious effect on actin cytoskeleton stability.

## Discussion

The present study used MEFs derived from *ROCK1^−/−^* and *ROCK2^−/−^* mice to investigate the role of ROCK isoforms in regulating ROS production and compare the effects of the genetic approach in combination with chemical antioxidant treatments on reducing doxorubicin-induced cytotoxicity. We found that both *ROCK1^−/−^* and *ROCK2^−/−^* MEFs exhibited reduced ROS production in response to doxorubicin treatment, but interestingly, only ROCK1 deficiency showed greater protection than antioxidant NAC including anti-ROS production, anti-apoptotic and anti-detachment effects. In addition, only ROCK1 deficiency showed synergistic protective effects when combined with NAC. Further examinations showed that *ROCK1^−/−^* MEFs exhibited reduced NADPH oxidase activation in doxorubicin treatment, which likely contributed to the diminished ROS. Moreover, ROCK1 deficiency not only enhanced the inhibitory effects of DPI on ROS generation and caspase 3 activation, but also attenuated cell detachment caused by DPI. The superior protective effects of ROCK1 deficiency over NAC, DPI treatments or ROCK2 deficiency were associated with the preservation of central stress fibers in response to doxorubicin, which was only observed in *ROCK1^−/−^* MEFs. These results support the concept that increased actin stability from ROCK1 deficiency sustains the protective effects of antioxidants, revealing a novel strategy to enhance cytoprotective effects of antioxidant treatments against cytotoxicity induced by doxorubicin which possibly can be extended to other oxidative stresses.

Many studies have suggested a chemo-protecting role for antioxidants [27], [28]. Nevertheless, various antioxidants allied with doxorubicin in clinical trials showed very limited beneficial effects against doxorubicin-induced cardiotoxicity and the cytotoxicity to other major organs [27],[28]. The present study provides a potential explanation for this below-expected outcome as antioxidants may have limited effects on cytoskeleton remodeling. Our results showed that although NAC or DPI treatment decreased ROS production and caspase activation induced by doxorubicin, NAC had no protective effect against the disruption of stress fibers, whereas DPI even increased the disruption of stress fibers. Although NAC treatment reduced MLC phosphorylation and actomyosin contraction, it also decreased cofilin phosphorylation leading to increased actin depolymerization. These inhibitory effects of NAC on both MLC and cofilin phosphorylation were previously observed in ROCK2 deficient cells, but not in ROCK1 deficient cells, in which only MLC phosphorylation but not cofilin phosphorylation was reduced. The simultaneous inhibition of MLC and cofilin phosphorylation by NAC is consistent with previous reports that antioxidant treatments reduce RhoA/ROCK activation [37], [38], which likely involve both ROCK1 and ROCK2. Together with our previous observations that ROCK1 deletion preserves actin cytoskeleton stability in response to doxorubicin or serum starvation [11], [12], the present study by combining NAC or DPI with doxorubicin further support the concept that ROCK1 deletion is highly effective in inhibiting actin cytoskeleton remodeling induced by cytotoxic stresses through preferentially inhibiting the cytotoxic pathway mediated by increased actomyosin contraction while preserving the cytoprotective pathway mediated by cofilin phosphorylation. It is worth noting that the increased cofilin phosphorylation leads to an increase in F-actin and decreases in G-actin, a process which may also cause cell function aberrant under some conditions. However, ROCK1 deletion has no detectable effect on F/G ratio at baseline condition [12]. Under stress conditions, especially cytotoxic stresses such as doxorubicin cytotoxicity or serum starvation [11], [12] which affect actin cytoskeleton stability, the increased cofilin phosphorylation by ROCK1 deletion has a beneficial role in maintaining F/G ratio and preserving actin cytoskeleton stability as well cell adhesion.

NADPH oxidase is one of the important ROS sources and is involved in doxorubicin cytotoxicity [32], [33]. We showed in this study that the inhibition of NADPH oxidase by DPI decreased ROS levels and caspase activation in doxorubicin treatment. We have also shown that ROCK1 deficiency reduced NADPH oxidase activity which was associated with reduced membrane translocations of p67phox and Rac1, which are key steps in the assembly of the active enzyme complex. The mechanisms underlying the inhibition of NADPH oxidase activation by ROCK1 deletion appears to be related with the reduced activation of Rac1 observed in the present study. Rac1 is an important subunit of NADPH oxidase and interacts with p67phox [35], [36]. Future studies are needed to investigate the mechanisms implicated in the inhibition of Rac1 activation by ROCK1 deletion and to explore other potential mechanisms involved in ROCK1-mediated NADPH oxidase activation in response to doxorubicin or other cytotoxic drugs. Previous reports have shown that ROCK was involved in the NADPH oxidase-mediated production of ROS in endothelial cells, hepatocytes and macrophages [39]–[41], but the contribution of each ROCK isoform was not determined. ROCK1 may be involved in the oxidative stress responses through modulating NADPH oxidase activity in a wide range of pathological conditions including inflammation, reperfusion injury and degeneration etc. It would be of interest to validate the present finding to other disease *in vitro* models and move forward to animal models of cardiovascular, metabolic and neurodegenerative diseases in which oxidative stresses play significant pathological roles.

Future studies are also needed to explore how the increased actin cytoskeleton stability from ROCK1 deletion enhances the anti-ROS, anti-apoptotic and anti-detachment effects of antioxidant treatments. Our previous studies have shown that the increased actin cytoskeleton stability from ROCK1 deletion promotes cell-cell and cell-matrix adhesion therefore increasing adhesion-mediated survival signaling and decreasing apoptosis [11], [12]. It remains to be determined whether the increased actin cytoskeleton stability from ROCK1 deletion results in reduced cellular energy consumption, and consequently contributes to reduced ROS production. It is worth noting that other potential sources of ROS overproduction induced by doxorubicin include mitochondria- and nitric oxide synthases-dependent ROS production [22]. It would be of interest to determine the effects of ROCK1 deletion on the ROS production from these sources. Finally, it would also be of interest to determine if combining ROCK1 deficiency with antioxidants only provides synergistic protective effects to normal cells, without reducing the tumor cell-killing ability of anti-tumor drugs including doxorubicin. In this regard, our recent study indicates that in contrast to normal cells, ROCK1 deletion reduces survival of oncogene-bearing cells, which exhibit different cytoskeletal organizations compared to normal cells [42].

**Figure 9:**
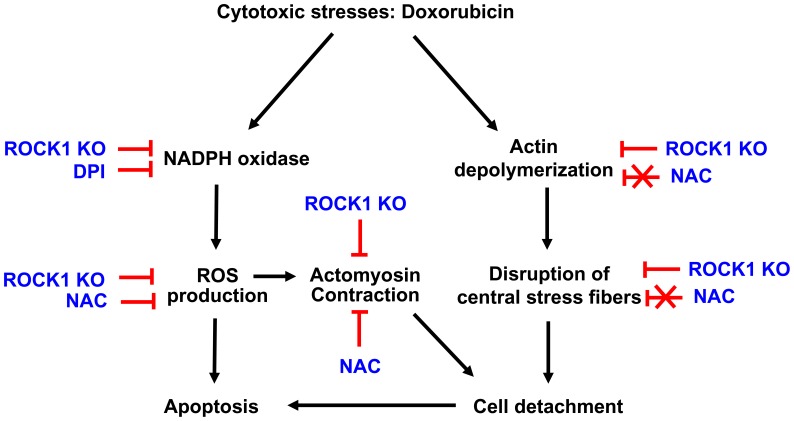
Schematic summary. The diagram summarizes the effects of anti-oxidants (NAC and DPI) and ROCK1 deficiency in opposing ROS production, apoptosis, actin cytoskeleton reorganization, and cell detachment induced by doxorubicin. NAC reduces ROS production leading to reduced apoptosis. NAC also decreases actin cytoskeleton remodeling in part through decreasing MLC phosphorylation and actomyosin contraction leading to reduced cell detachment. However, NAC inhibits actin polymerization through reducing cofilin phosphorylation, and therefore does not prevent the disruption of central stress fibers. DPI reduces ROS production and apoptosis, supporting a contributory role in doxorubicin-induced ROS production. ROCK1 deficiency reduces ROS production, in part through inhibiting NADPH oxidase activation. Finally, ROCK1 deficiency presents a unique property of preserving central stress fiber stability through reducing MLC phosphorylation and preserving cofilin phosphorylation, leading to the preserved cell adhesion which then enhances the protective effects of antioxidant treatment against doxorubicin-induced ROS production, apoptosis and cell detachment.

In summary, this study indicates that targeting the ROCK1 pathway significantly enhances the protective effects of antioxidant treatments against doxorubicin cytotoxicity, providing a novel strategy for chemoprotection. ROCK1 deficiency improves antioxidant treatment through multiple mechanisms including reducing NADPH oxidase activation and ROS production as well as preserving actin cytoskeleton stability in response to doxorubicin. A model to summarize all of these findings is schemed in Fig. 9. Future studies in animal models are needed to validate this novel strategy for chemoprotection against doxorubicin and other chemotherapeutic drug-induced oxidative stress and toxicity. During the last decade, the ROCK family has emerged as a promising therapeutic target in a wide range of human diseases, including cardiovascular disorders, neurologic disorders, metabolic disorders, and cancers [43]–[46]. The currently available ROCK pan-inhibitors produced beneficial effects in many experimental and clinical studies, in particular in a mouse model of doxorubicin-induced chronic cardiotoxicity [47]. However these inhibitors inhibit both ROCK1 and ROCK2, and destabilize actin cytoskeleton to a worse degree than the outcome of the ROCK2 deletion [11]; they may not enhance protective effects of antioxidants against doxorubicin and other oxidative stress-related cytotoxicity, and may even apply cytotoxic effects. It is worth noting that the inhibition of ROCK1 deletion on actin cytoskeleton remodeling observed in this study is consistent with our previous *in vivo* observations that ROCK1 deletion inhibits cardiac remodeling and cardiomyocyte apoptosis in pathological heart failure [48]–[50]. Future studies are needed to determine if ROCK1 deficiency not only reduces aberrant contraction through decreasing MLC phosphorylation in the vasculature and in the heart in response to pathological stimulation, but also improves survival of cardiovascular cells, especially endothelial cells and cardiomyocytes, through preserving actin cytoskeleton stability in these cells, which are the major targets of pathological stimuli including doxorubicin-induced cardiotoxicity. The current observations therefore support the development of isoform-selective ROCK1 inhibitors.

## Materials and Methods

### Cell Culture and Treatments

ROCK1- or ROCK2-deficient MEF cells were prepared from *ROCK1* ^−/−^ or *ROCK2* ^−/−^ embryos as previously described [11]. All animal experiments were conducted in accordance with the National Institutes of Health “Guide for the Care and Use of Laboratory Animals” (NIH Publication No. 85-23, revised 1996) and were approved by the Institutional Animal Care and Use Committee at Indiana University School of Medicine. Cells were cultured in Dulbecco’s modified Eagle’s medium (DMEM; Life Technologies) supplemented with 10% fetal bovine serum (FBS; Atlanta Biologicals) and penicillin-streptomycin in a humidified incubator with 5% CO_2_ at 37°C. Cells at 80–90% confluency were treated with various drugs at indicated times and dosages. These drugs include doxorubicin, NAC and DPI (Sigma).

### Cell Viability and Detachment Assays

For cell viability assay, following the treatment with desired drugs at indicated concentrations and time points, methylthiazole tetrazolium (MTT) assay was performed as previously described [11]. At least three independent experiments were analyzed. For the cell detachment assay, cells were cultured in DMEM supplemented with 10% FBS to reach 90% confluency, followed by the treatment of desired drugs at indicated concentrations and time points. Detached cells in culture medium (floating cells) were collected at indicated times and counted with a hemacytometer. The attached cells were harvested by trypsinization for counting. Cell viability of floating and attached cells was also determined by assessing cellular uptake of trypan blue dye as previously described [11]. At least three independent experiments were analyzed for each condition.

### Fluorescence Imaging

Phalloidin staining of F-actin and immunofluorescence staining of p-MLC staining were performed as previously described [11]. To detect ROS in live cells, CM-H2DCFDA (C-6827, Life Technologies) was used as a reliable fluorogenic marker of ROS in cells, while 4′,6-diamidino-2-phenylindole (DAPI) was used for nucleus staining; both reagents are membrane permeable in live cells. MEF cells were seeded and attached to gelatin coated glass coverslips. After treating cells with desired drugs at indicate time points, CM-H2DCFDA was added to culture medium at a final concentration of 13 µM, and incubated for 10 min at 37°C. Cells were then washed three times with pre-warmed phosphate-buffered saline (PBS). Coverslips were then mounted in with Vectashield mounting media (H-1200, Vector Laboratories) containing DAPI. The oxidative stressed and non-stressed cells were able to be distinguished under fluorescence microscopy (Leica DM5500B with objectives: HCX PL FUOTAR 20.0×0.50, HCX PL FUOTAR 40×0.75) equipped with a DFC300FXR2 camera, and images were analyzed with the Leica AF6000 software.

### ROS Measurement by Flow Cytometry

After treating MEF cells with desired drugs for indicated time periods, medium was removed and cells were incubated in PBS for 10 min, followed by 30 min incubation at 37°C in DMEM without phenol but containing 5 µM of CM-H2DCFDA. The attached cells were collected and suspended in PBS buffer for flow cytometry analysis. All data were acquired with Becton Dickinson FACSCalibur and analyzed with CellQuest software. The samples under the same treatment were prepared in triplicate, and the data were acquired twice by FACS.

### Protein Analysis

Following treatment with desired drugs, attached cells were harvested and analyzed for caspase activation by Western blot analysis as previously described [11]. The blots were then probed with primary antibodies to ROCK1 (GTX61382) from GeneTex, ROCK2 (sc-5561) from Santa Cruz Biotechnology, p67phox (07-502) from Millipore, cleaved caspase 3 (#9661), cleaved caspase 9 (#9509), cleaved caspase 8 (#9429), PARP (#9542), cofilin (#3312), p-cofilin(Ser3) (#3311), MLC (#3672), p-MLC(Ser19) (#3671) from Cell Signaling. After blotting with corresponding secondary antibodies conjugated with horseradish peroxidase, the membranes were developed with ECL Western blotting or SuperSignal West Pico Chemiluminescent Substrate (Thermo Scientific), and the blots were visualized by using a Fujifilm LAS-4000 Imager. All blots were normalized to GAPDH (MA5-15738, Thermo Scientific) or to actin (sc-1616; Santa Cruz Biotechnology).

### Subcellular Fractionation

To separate cytosolic and membrane fractions, cells were washed with cold PBS after removing culture medium, solubilized with Tris lysis buffer (20 mM Tris, pH 7.5, 100 mM NaCl, 5 mM EDTA plus protease inhibitor and phosphatase inhibitor cocktails (Roche)). The debris was pelleted at 700 *g* for 10 minutes, and the supernatant was further centrifuged at 100,000 *g* for 60 minutes at 4°C. The supernatant then was saved as the cytosolic fraction and the pellet was suspended in Tris lysis buffer containing 1% Triton X-100 as the membrane fraction. Proteins (50 µg) from each fraction were analyzed by Western blotting as described above.

### NADPH Oxidase Activity Assay

NADPH oxidase activity in intact cells was measured using a modified assay [51]. Briefly, photon emission from the chromogenic substrate lucigenin was measured every 15 s for 20 min in an Lmax Microplate Luminometer (Molecular Devices). Lucigenin serves as the acceptor of electron/O_2_^−^ generated by the NADPH oxidase complex. Activity measurement was performed in a 250-mM HEPES buffer, pH 7.4, containing NaCl 120 mM, KCl 5.9 mM, MgSO_4_ 1.2 mM, CaCl_2_ 1.75 mM, EDTA 0.5 mM, glucose 11 mM, lucigenin 0.2 mM, and NADPH 0.1 mM as the substrate. Non-treated or doxorubicin-treated cells were collected, counted, and pelleted at 400×*g* at 4°C for 4 min; the pellet was re-suspended in a balanced salt solution containing NaCl 130 mM, KCl 5 mM, MgCl_2_ 1 mM, CaCl_2_ 1.5 mM, phosphoric acid 35 mM, and HEPES 20 mM, pH 7.4. 0.5×10^6^ cells in balanced salt solution containing 10 mM glucose and 1 mg/mL bovine serum albumin were used in measurement. A buffer blank was subtracted from each reading before data calculation. No activity could be measured in the absence of NADPH or in the presence of 100 µM DPI.

### Rac1 Activity Assay

Rac1 activation was assessed by Rac1-GTP pull down assay using PAK-1 PBD-agarose beads (#17–283, Millipore). Cell lysates (800 µg) were pre-cleared with glutathione agarose beads (SC-2009, Santa Cruz)) at 4°C for 10 min. The supernatant was then incubated with PAK-1 PBD-agarose beads at 4°C for 1 h. The beads were then washed 3 times with the lysis buffer and followed by boiling in Laemmli reducing sample buffer to release active Rac1, which was detected with anti-Rac1 mouse monoclonal 23A8 (05–389, Millipore) by Western blotting.

### Statistical Analysis

Data are reported as mean ± SE. Comparisons between groups were analyzed by Student’s *t*-test or ANOVA as appropriate, with *P*<0.05 considered as significant.
